# Semi-automatic identification of punching areas for tissue microarray building: the tubular breast cancer pilot study

**DOI:** 10.1186/1471-2105-11-566

**Published:** 2010-11-18

**Authors:** Federica Viti, Ivan Merelli, Mieke Timmermans, Michael den Bakker, Francesco Beltrame, Peter Riegman, Luciano Milanesi

**Affiliations:** 1 Institute for Biomedical Technologies of the National Research Council, Segrate (Milan), Italy; 2 Department of Pathology of the Josephine Nefkens Institute, Erasmus Medical Center, Rotterdam, The Netherlands; 3 University of Genoa, Department of of Communication Computer and System Sciences, Genoa, Italy

## Abstract

**Background:**

Tissue MicroArray technology aims to perform immunohistochemical staining on hundreds of different tissue samples simultaneously. It allows faster analysis, considerably reducing costs incurred in staining. A time consuming phase of the methodology is the selection of tissue areas within paraffin blocks: no utilities have been developed for the identification of areas to be punched from the donor block and assembled in the recipient block.

**Results:**

The presented work supports, in the specific case of a primary subtype of breast cancer (tubular breast cancer), the semi-automatic discrimination and localization between normal and pathological regions within the tissues. The diagnosis is performed by analysing specific morphological features of the sample such as the absence of a double layer of cells around the lumen and the decay of a regular glands-and-lobules structure. These features are analysed using an algorithm which performs the extraction of morphological parameters from images and compares them to experimentally validated threshold values. Results are satisfactory since in most of the cases the automatic diagnosis matches the response of the pathologists. In particular, on a total of 1296 sub-images showing normal and pathological areas of breast specimens, algorithm accuracy, sensitivity and specificity are respectively 89%, 84% and 94%.

**Conclusions:**

The proposed work is a first attempt to demonstrate that automation in the Tissue MicroArray field is feasible and it can represent an important tool for scientists to cope with this high-throughput technique.

## Background

**Figure 1:**
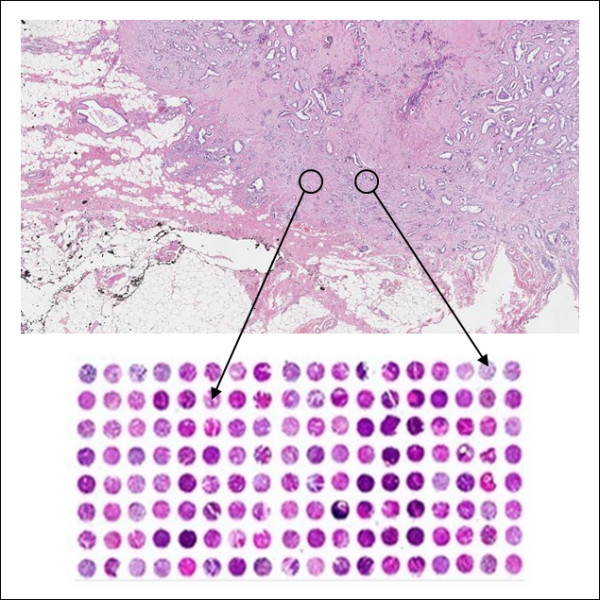
**TMA**. Top: H&E stained slide of the tissue with marks to highlight punching areas. Bottom: the recipient block.

One of the most powerful high-throughput histology related analysis applications of recent years in medical research is the Tissue MicroArray (TMA) technique [1]. This technique is commonly used for many medical research projects and is well recognized, among others, in the large scale Human Protein Atlas project [2]. It is based on the use of formalin-fixed paraffin-embedded (FFPE) tissue samples, designated donor blocks. Cores from the donor blocks are organized as a matrix in a new paraffin recipient block, the TMA (Figure 1). The array design and content can be highly customized, according to the aim of the experiments, and may be based on phenotype or genotype features, usually to examine differences among diseases, or to study the effect of drugs on tissues.

The intrinsic high parallelism of a TMA block, due to the matrix structure that allows the examination of many different tissues at the same time, leads to several advantages, such as a decrease in the time taken to perform one assay instead of hundreds of separate assays, and a limited reagents cost, as around 500 separate experiments can be completed on a single TMA. On the other hand the technique presents particular challenges in the input/output data evaluation, including the investigation of the results of hundreds of different tissues at the same time. In addition, TMA construction is highly time-consuming because it requires a pathologist to identify, on every donor block, the area suitable to be punched-out and inserted into the array block. This aspect is often underestimated.

Since the technique is relatively new, up to now few specific software tools have been developed to support TMA construction. To carry out image evaluation, scientists require specific support in two important phases. First, in the evaluation of hematoxylin-eosin (H&E) stained slides of donor block tissues (pre-array images), to highlight the interesting areas for TMA building. This is a highly time consuming task and in most pathology institutes evaluations are performed completely by hand for each of the hundreds of tissues that will compose the matrix. Secondly, the scoring phase on TMA samples (post-array images) may be improved by the use of automatic algorithms. Considering that a single TMA block may theoretically yield around a hundred of stained sections and that each section may contain hundreds of samples (cores), the need for support tools is evident. Therefore, it is clear how the impact of the TMA technique may be significantly enhanced by suitable and efficient algorithms to analyse tissue images.

**Figure 2:**
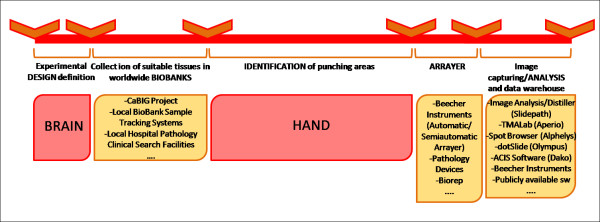
**Experiment timeline**. Overview of the whole TMA experiment process associated to some of the best known tools now available.

To accomplish this task a number of applications exist. Some software tools are commercially available and generally these allow the user to capture spots in images, organize them into a sorted matrix, annotate and analyse them with quantitative imaging, including nuclei counting and signal quantification or some morphology measurements. In addition, for most of these solutions the image software cannot be released as standalone and is usually distributed together with a virtual microscope system. However, these software packages focus on the final analysis phase and are not suitable for aiding automation in the pre-array phase. Moreover, they are not very flexible for keeping up with ongoing experimental research and they have not been developed for automatically identifying variations in morphologies, which is the crucial feature in the pre-array phase. The most used tools, listed in Figure 2, are provided among others by Slidepath, Aperio, Olympus, Dako and Beecher Instruments.

Useful highly customizable software can be found as open source applications [3-5]. Most of these rely on web interfaces running on top of local databases to store TMA related information, such as patients' clinical data, specimens, donor blocks, cores, and recipient blocks and in some cases also provide the possibility to digitally process microscope acquired images.

All of these packages take into account the post-array images, used in the analysis phase, providing semi-quantitative evaluation tools for the stained core images and assigning to them reaction scores, or exploiting extracted parameters to generate statistical results. Nevertheless the identification and localization of suitable areas for extracting tissue cores that are to be used for TMA construction represent a major bottleneck in a TMA experiment.

In this paper we present an algorithm for efficient TMA construction by automatic identification of areas of interest in a TMA donor block, which has been included in the TMARepDB [5] web site. In the pre-array phase, the identification of areas that are suitable for extracting the donor cores is not yet covered by any TMA-oriented specific automation. This would require software to distinguish normal and pathological morphologies within a tissue. In this context the biological complexity of human tissues represents a challenge. Moreover, matters are complicated by the fact that cancer is, by definition, an abnormal and uncontrolled proliferation of cells, further increasing the complexity of the histology. This means that the often heterogeneous areas must be evaluated with different features, and consequently the use of independent algorithms may be required. Through a pilot study on tubular breast cancer, the paper shows the feasibility of creating an integrated system to deal with the peculiarities of each different tissue found in donor block sections. The focus of the work is the development of an algorithm able to analyse breast tissue images which provide useful representations of routinely prepared H&E stained sections of tubular breast cancer regions enabling a pre-selection of suitable areas for TMA construction.

Other works exist which describe automatic approaches to H&E stained tissue slides, to support specifically the field of histopathology and to improve the potentiality of telepathology and virtual pathology. An interesting approach for detecting cancers from digital tissue images has been proposed [6] concerning the identification of the Gleason grade, again in the context of prostate tissues. The main steps of the pipeline concern a Bayesian classifier to detect gland lumen and the use of a Support Vector Machine (SVM) to classify each tissue pathology grade. Another work [7] has been proposed to deal with the automatic detection of head and neck squamous cell carcinoma: a machine learning approach has been used, with the development of a SVM classifier. Image pre-processing has been performed exploiting the density based spatial clustering of applications with the noise (DBSCAN) method [8]. Nevertheless, the SVM machine learning approach does not always represent the best choice, because, in our opinion, when investigated features are well defined more robust solutions can be adopted.

For analysing breast tissue, two state-of-the-art algorithms are presented [9,10]. Both methods implement the three levels of the Nottingham system, which is a scoring method that uses the range 3-9 to assess the grade of breast cancers. The former is based on Otsu segmentation method [11] and the use of thresholds to detect and score tubule formation, nuclear pleomorphism and mitotic cells, which contribute to the final grading. The latter evaluates two discriminant parameters, the number density of cell nuclei with disperse chromatin and the number density of tubular cross sections, through the exploitation of the LNKnet tool [12], developed at the MIT (Massachusetts Institute of Technology). Nothing exists in the specific context of tubular breast cancer.

The MAGIC system [13] represents an application in anatomo-pathology field of the Baatz and Schape method [14], a general purpose image processing for high quality multi-scale segmentation. The implemented system is aimed at detecting histological objects in the prostate tissues by exploiting fully automatic tools for segmentation, classification, and extraction of color, texture, and morphometric features. The exploited method allows the accurate identification of histological classes of stroma, nuclei (epithelial, apoptotic, and stroma), cytoplasm, red blood cells, and lumens. This is a valuable system, also enriched by a graphical user interface, with the limitation of being only commercially available. Moreover, as many other digital pathology works, it is oriented to H&E images analysis more than to tissue areas localization for pre-array punching purposes.

Even other valuable commercial software exists, mainly oriented to tissue analysis in TMA and the pathology analysis context: among them the tools implemented by TissueGnostics [15], Beecher Instruments [16] and Aperio [17] Companies.

## Biological context

Tubular carcinoma was chosen as pilot subject for the development of the presented tool. It is a type of invasive ductal carcinoma of the breast. It takes its name from its microscopic appearance, in which the cancer cells resemble small tubes. In some cases, tubular cancer cells are mixed with ductal or lobular cancer cells, giving a mixed-tumor diagnosis. Tubular carcinomas are often very small, but may show up on a mammogram, as an irregularly shaped mass with a spiky, or starry outline.

It shows morphological features that differentiate it from other types of breast cancer, and that can be used to define parameters for developing an automatic approach for its recognition.

**Figure 3:**
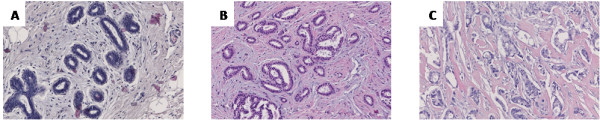
**Tissue morphologies**. Normal (A), single layer pathological cells (B) and random distributed pathological cells (C) breast tissue images.

The first feature that has been considered is the presence of a single layer of cells around the lumen of the glands (Figure 3B). In normal breast tissue each lumen is surrounded by two concentric layers of cells creating the acini of the lobules, as shown in Figure 3A. The second characteristic of tubular breast carcinoma is the random organization of the tubular structures without the typical breast morphology of glands-and-lobules (Figure 3C).

## Methods

### Dataset

The algorithm has been developed using a primary set of 20 tubular breast cancer biopsies provided by the Erasmus MC tissue bank hosted at the Department of Pathology of the Erasmus MC in Rotterdam (NL). Standard 4 *μ*m H&E sections were prepared from the FFPE tissue blocks and digitalized using a Virtual Microscope (VM; Nanozoomer, Hamamatsu, Japan) [18], which allows the acquisition of the whole slide at high magnification through different available virtual objectives. The images were all acquired using a magnification of 200*X *and having the same dimension (6720 × 4200). All the H&E images have been valued by two experts in tissues morphology recognition, which discriminate pathological regions from normal ones. In case of contrasting evaluation, which occurs in about 10% of the analysis, the corresponding images have been removed from the dataset. To allow a suitable sampling of the tissue (coherently with the pathologists defined sampling criterion) and according to the defined magnification and dimension, the algorithm automatically subdivided these images using a 8 × 9 punching grid which results in a reference dataset composed by 1296 sub-images. Using a classical approach for classification problems, the whole dataset has been separated into three parts: the training set, the tuning set and the validation set. The first set, composed of around 50% of the available sub-images (650), is used to build the model, in particular to evaluate the parameters suitable for the pipeline implementation; the second set, composed of 25% (323) of the available sub-images, is exploited to tune the model; the third set, composed of 25% (323) of the available sub-images, is necessary to measure the performance of the algorithm, holding the identified parameters as constant values. In order to guarantee the reliability of the results a cross-validation is performed, by repeating the classification four times, randomly selecting the images belonging to each group. During each step of the crossvalidation test parameters have been optimized in order to achieve the best possible results. Remarkably, considering for each algorithm parameter the interval in which best values are placed for each experiment the range is quite narrow and the variance quite low. In agreement to the achieved results the algorithm default parameters have been selected according to the experiment that provided the best performance in terms of accuracy: data are reported in the following sections. In order to test the effective flexibility of our algorithm, two more datasets of images originated with different resolution and different pixel intensity have been exploited. One dataset includes 5 images presenting a resolution of 0.23 *μm*/*px *and a pixel intensity similar to the reference dataset. According to our system, images from this dataset are converted in the preliminary algorithm step in order to obtain images presenting 200*X *of magnification and 0.46 *μm*/*px *of resolution (considered in this work as *R_REF _*, the reference value for resolution, but customizable by the user to better suit different digitalization features).

The other dataset includes 5 images showing the same resolution of the reference dataset but different pixel intensity. The first step when analyzing these images consists in normalizing to the average pixel intensity of the training set *P_REF _*= 170 (also customizable by the user for finely tuning the algorithm). For both sets, images that encountered contrasting tissue evaluation among pathologists (around 10% of the whole sets) have been removed from the datasets. As reported in the performance section, prediction capabilities of our algorithm was good in comparison with the primary dataset (for which this algorithm has been specifically designed). In both cases by providing a fine-tuning of the algorithm parameters (e.g. setting the effective resolution for the first dataset and the effective pixel intensity for the second dataset) the performance showed improvements.

### Algorithms

**Figure 4:**
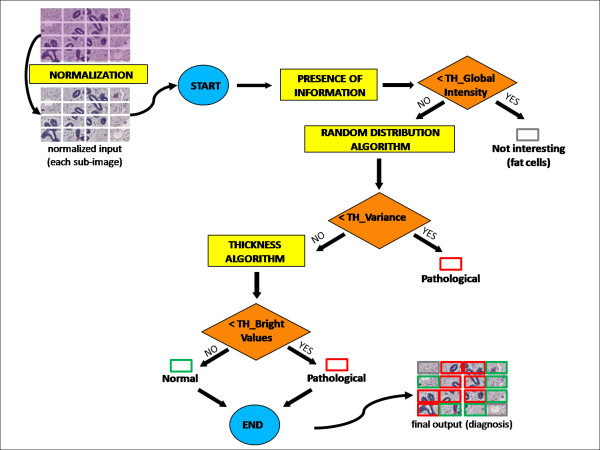
**Algorithm workflow**. Simplified work flow of the developed pipeline.

The workflow for the identification of pathological regions is presented in Figure 4. It has been designed and evaluated in collaboration with biologists and pathologists, who deal with H&E slides analysis and manual biopsies diagnosis on a daily basis.

As a preliminary step, each whole image of an H&E stained section acquired through a virtual microscope is normalized with respect to image intensity and magnification, in order to make the images comparable to each other and thus providing algorithm thresholds suitable for all the considered images. Each image has then been scaled to specific reference pixel intensity values (*B_R _*for background, *N_R _*for nuclei, *C_R _*for cytoplasm and their average value *P_REF _*), in order to eliminate the differences in brightness among images acquired at different times or with different instruments. The transformation is performed according to a well-established colour normalization approach [19] which relies on the evaluation of three specific areas to take as references for a quadratic scaling. In H&E staining context key values are identified as the average intensities of background, nuclei area and cytoplasm regions. For each whole digital slide, these colour-levels have been extracted through segmentation methods and related histograms evaluations, and were then used for data calibration. Pre-defined values have been assumed as references. A quadratic mathematical transfer function is used to get a new set of normalized images. For each image, the calibration coefficients A, B, C are automatically calculated based on the areas equalization:

(1) $$\{\begin{array}{c} y_{i,(B_{R})}=Ax_{i,(B_{N})}^{2}+Bx_{i,(B_{N})}+C\\ y_{i,(N_{R})}=Ax_{i,(N_{N})}^{2}+Bx_{i,(N_{N})}+C\\ y_{i,(C_{R})}=Ax_{i,(C_{N})}^{2}+Bx_{i,(C_{N})}+C \end{array}$$

where *x_i _*and *y_i _*correspond to the value of the i-pixel of the image, respectively before and after the calibration on the reference values. In particular:

*x_i_*,(*B_N _*) represents the background average value of the image to be calibrated

*y_i_*,(*B_R_*) represents the background average value of reference image;

*x_i_*,(*N_N_*) represents the nuclei area average value of the image to be calibrated

*y_i_*,(*N_R_*) represents the nuclei area average value of reference image;

*x_i_*,(*C_N_*) represents the cytoplasm area average value of the image to be calibrated

*y_i _*,(*C_R_*) represents the cytoplasm area average value of reference image.

For each image, A, B, and C transfer coefficients are automatically calculated based on the previous data. All pixels of images were mathematically converted according to the mathematical transfer function and after the standardization step, images of the same dataset showed the same average colour levels on the background area, on the nuclei regions and on the cytoplasm areas.

When dealing with resolution normalization, images were always scaled to the reference value *R_REF _*. It must be noticed that the user can intervene on the image normalization phase, in order to better adapt the software to manage images acquired through different technologies, by providing both customized *R_REF _*and *P_REF _*values before running the algorithm. This fine-tuning possibility allows the user to redefine the default values coming from the dataset on which this work relies, since performance could slightly suffer the preliminary conversion step of the algorithm. Concerning *R_REF _*a simple re-dimensioning is performed, while regarding *P_REF _*, even new values of *B_R_*, *N_R _*and *C_R _*components are calculated, maintaining the same proportions existing in the original dataset.

Before enabling image classification using the designed algorithm, the image is divided into a number of independent sub-images, to be individually analysed and labelled according to the result of the computed diagnosis: the generated grid allows a tissue division where from each area corresponding to one sub-image one tissue punching can be performed randomly.

The successive step of the algorithm consists in discovering if either (a) the image contains significant information for the segregation of un-affected and affected areas, or (b) it represents an area where no tissue is present at all, or the tissue does not have the characteristics of affected or unaffected tissue (i.e. fat cells). To perform this classification the average intensity of each image is calculated and compared with a threshold value. If the image is consistent with case (a), the algorithm must proceed, if the image is consistent with case (b) the algorithm stops. If the selected image is labelled as informative, the work flow evaluates the first discriminating feature among normal and pathological tissue, which is the random distribution of cells. The basic idea for performing the step arises by observing the whole tissue organization: in affected areas cell agglomerates are randomly distributed within the tissue and are much smaller and more numerous than in unaffected areas. Therefore, the designed algorithm relies on object detection and area estimation: areas values are successively evaluated in order to distinguish if they belong to a well organized or a random distributed structure. At first a pre-processing step is required: each image is filtered in a suitable way to obtain a black and white mask where only gland nuclei are highlighted. Firstly tissue components had to be separated virtually. A robust and largely used method for performing tissue image segmentation [20-23] is k-means clustering, an unsupervised algorithm used to group *n *objects based on attributes into *k *partitions (*k *<*n*).

Practically, k-means clustering takes pixel intensity values as inputs and considers three random values as starting points. Using the Euclidean distance it creates three groups that include pixels whose intensity values are close to the three values chosen as starting points. For each group the centroid is calculated, and the three groups are modified considering the centroids as the new starting points.

By applying this procedure to the H&E image and by iterating it, the algorithm approaches the local minimum of the function. When the group variance converges to the minimum each pixel group that has one distinct class label represents one segmented object component. In the studied context it separates the three main different colours of the image generating three result images:

• the white based image, showing the mucus presence

• the pink based image, indicating the basic areas (such as cytoplasm)

• the blue based image, reporting the acid regions (such as nuclei)

This method even enables the isolation of the dark blue intensities that characterize nucleic substance images. This is possible by ranking the pixel values by their intensity and just considering the darker ones, that correspond to nuclei, which are the most highly acid substance within the cell. Such a processed image is then converted from an RGB (Red-Green-Blue) map to gray-scale and the agglomerate shape is highlighted using two morphological filters: dilation and hole filling.

After the pre-processing phase, area detection is performed by including each unknown-shaped agglomerate into fitting ellipses, defined by their semimajor and semiminor axes. If the analysed sub-image can be classified as random-distributed pathological tissue in this step, the work flow comes to an end and labels the image as affected.

If the work flow does not reach an endpoint the presence of a double or a single layer of cells is investigated, using a *thickness *algorithm based on Hildebrand and Ruegsegger approach [24]. The choice of this algorithm depends on the fact that it is a model independent thickness estimator, which is a very important feature since it has to deal with objects with an a priori unknown structure type. Following this approach local widths are obtained by virtually inserting a disk into each image object and identifying the largest disk completely contained within the object structure. Analytically, the local thickness τ (*p*), where *p *is an arbitrary point in the structure Ω ⊂ *R*^3^, can be defined as:

(5) τ(p)=2max(r|p∈sph(x,r)⊆Ω,x∈Ω)

where *sph*(*x, r*) is the set of points inside a sphere with center *x *and radius *r*. The maximum local thickness is equivalent to the diameter of the largest sphere that completely ts inside the structure:

(6) $$\tau_{max}=max(\tau (p)|p\in \Omega )$$

The algorithm works on the segmented image, by creating a distance map which assigns the Euclidean distance to the nearest edge point to every point within each segmented object. This is equivalent to the radius of the largest sphere centred at the considered point and still completely inside the structure. The inclusion tests lead to the identification of non-redundant spheres, that will define the distance ridge. The thickness algorithm provides a false-colour image, which for each point shows the thickness of the structures by assigning different colours to disks of different diameter: in particular brighter colours are associated to larger disks. The output of the phase is the separation between single-layer pathological tissue and normal tissue. Each sub-image is analysed with the described work flow, giving as final output a diagnosis of unaffected, affected, or fat area, indicated by the colour of the border that is overlaid on the original image in the last step of the analysis. The red border means that the considered area is affected, the green border designates unaffected areas. Both the green and the red areas may be of importance for selecting cores for the TMA. The grey border represents areas without tissue or tissue that is mainly fat. Since each image is only a part of the whole slide, in the final step the image is reassembled, thus providing a view that allows the correct choice of the areas to be punched for building the TMA block. Labelled areas may differ from one region to another, due to the complexity of the tumour tissue mixed with normal tissue. The two biological aspects described, the layers of cells forming the lumen and the morphological organization of the tubular structures, were first considered in separate algorithms, and then combined in the unified work flow.

### Implementation

The algorithm has been implemented using Perl language, embedding external functions based on different software, among which Matlab [25], a commercially available computing environment which provides a dedicated toolbox for image analysis (Image Processing Toolbox), ImageJ [26], a Java based open source application for image management, particularly devoted to bio-images, and command line executables distributed in the ImageMagick GPL licensed package [27].

## Results and Discussion

### Threshold values identification

A crucial role for a successful application of this algorithm is the selection of the parameters that characterize the examined features and the related threshold values for decision making. In the following section the whole work flow will be retraced in order to analyse the parameters used for each conditional step.

#### Pre-processing

Pre-processing step includes, other than the normalization phase, the detection of the correct sub-image size, and the identification of a suitable grid to cut the original image. The sub-image dimension is strictly connected to the aim of the proposed work, that is to select punching areas for Tissue Microarray experiments. Therefore, the grid spacing and the sub image size cannot be arbitrarily varied. It depends on image resolution *R_REF _*and is defined in order to guarantee a correct tile size with respect to real biological object size: the grid must be large enough to provide morphological consistency within each tile, and thick enough to always allow the correct distance between the selected punching areas.

#### Not interesting sub-images detection

Concerning the first phase, related to the presence of interesting information inside the considered sub-image, the discriminating factor is identified in the average value of the pixel intensity of the sub-image itself. The threshold value to distinguish between interesting and not interesting sub-images is set to an intensity value identified by analysing a training set of images including significant, void and fat areas. The value correspondent to the intensity that discriminates between brighter and darker images, thus defining those that do not contain informative tissue areas, is 1.2*P_REF _.

#### Cells distribution evaluation

**Figure 5:**
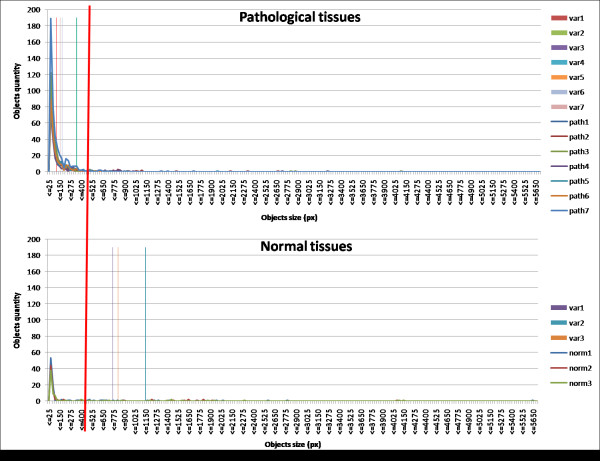
**Cell distribution evaluation**. Results from the randomness evaluation step. Above plot: distribution of ellipse areas (representing biological object areas) in pathological tissue images. On the x-axis the distribution of objects area values is plotted; the y-axis shows the number of objects presenting the same area. Seven different sub-images are considered (line graphs) reported in the side legend, together with signals of standard deviations (singletons). Below plot: distribution of ellipse areas (representing biological object areas) in morphologically well structured tissue images. On the x-axis the distribution of objects area values is plotted; the y-axis shows the number of objects presenting the same area. Three different sub-images are considered (line graphs) reported in the side legend, together with signals of standard deviations (singletons). The red line crossing both plots represents the chosen threshold, which clearly separates among areas distributions retrieved from normal and pathological tissues.

Proceeding with the model investigation, from empirically analysing results achieved on the training set the most reliable parameter for the analysis of the cell distribution appears to be the standard deviation of ellipse area values. When plotting this data in *X *- *Y *axis a significant difference is observed between tissues with random organization of elements and tissue with the characteristic breast tissue structure. Figure 5 shows this difference in a common reference scale.

To avoid false positive results which may be caused by the presence of single T and B lymphocytes, which are frequently seen in inflamed tissues but that are not specifically involved in tumour proliferation, area values lower than a fixed threshold were rejected before plotting the data. This size filter has been established to $1/R_{REF}^{2}*64$, which is consistent with the dimension of a typical lymphocyte (around 8 micron) on a digital image.

The considered signal is represented by the distribution of the ellipses areas within an image. The signal standard deviation is computed and, repeating the process for each image of the training set, a biologically consistent value of this parameter has been obtained, which distinguishes between the well organized and the random distributed tissues, thus representing a threshold value. This discriminating value is set to $1/R_{REF}^{2}*95$, which well distinguishes among normal images, presenting a higher variance of the object areas, and pathological ones, correspondent to lower variances.

#### Cell layers thickness investigation

**Figure 6:**
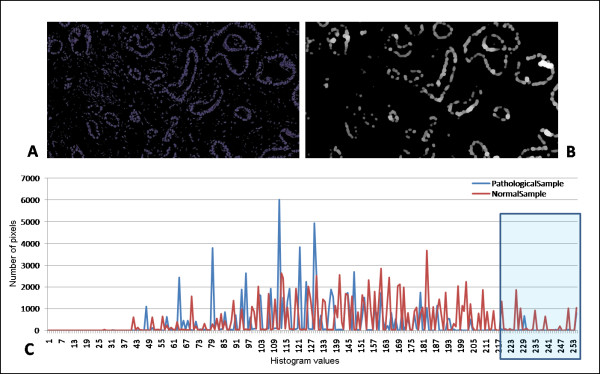
**Cell layers estimation**. Example of the typical sub-results from thickness evaluation step. (A) Color segmentation to separate nuclei from the rest of the image. (B) The black and white mask generated by Hildebrand and Ruegsegger algorithm. (C) Histogram of the image intensity, showing the number of pixels counted for each histogram bin in both normal (red) and pathological (blue) example cases: it can be noticed that in the normal sample the pixels corresponding to higher intensity are more numerous than in the pathological sample, due to the presence of thicker structures within physiological tissues.

To answer the final conditional block of the work flow, concerning the thickness of the cell layer around the lumen, a histogram profile is produced for each image resulting from the thickness algorithm application. Converting the image from RGB to gray scale, major thickness, related to unaffected double-cell surrounded structures, are dyed in bright colours, while thin objects, mostly present in pathological tissues, show dark colours. In fact, histogram comparison showed differences between the normal and the pathological tissues especially concerning the highest values of intensity, as expected. In this case the analytical parameter chosen to express the observed differences is the sum of the quantity of pixels estimated for brightest colours (experimentally set to intensities ≥1.3_* _*P_REF _*) in the histogram plot. In fact, according to given representation of the Hildebrand and Ruegsegger, higher intensity values correspond to major thickness of image objects. The value of this parameter in unaffected areas is always higher than in pathological ones, as shown in Figure 6.

A descriptive statistical evaluation has been performed on all data generated from the training set images to infer a parameter useful to distinguish physiological from pathological areas. The quantity of bright pixels within an image has been found suitable to discriminate among normal and pathological regions. The value has been identified, which depends on the *R_REF _*: images presenting more than $1/R_{REF}^{2}*530$ bright intensity pixels include thick objects within the tissue while images with an inferior number of bright pixels are representative of just one layer of cells around the lumen.

### Performance

**Figure 7:**
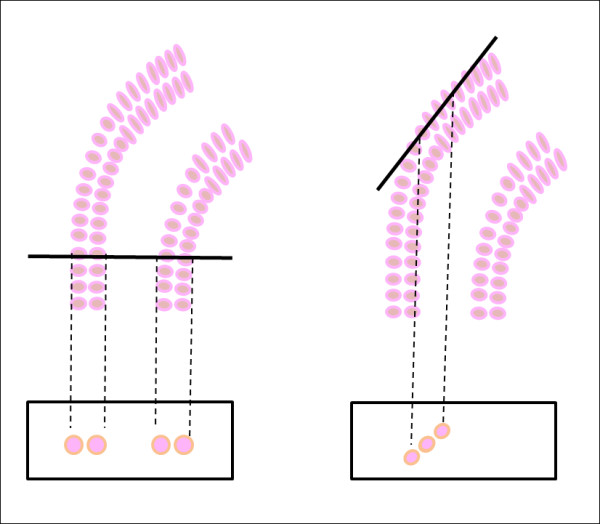
**Cutting plane**. Different cutting directions over tissues can output diverse information on images: the two layers can be well shown (A) or represented data can be misleading (B).

The results performances have been evaluated considering the judgment of two experts, to provide accuracy in histological assessment, in order to reduce any potential intra-observer variability [28]. Concerning the primary dataset of biopsies, algorithm performances are summarized in Table 1. Data are reported according to the four performed cross-validations, showing the experiments outcome and the related accuracy. The optimal algorithm parameters have very low variability among the different experiments, which can be attributed to the intrinsic physical meaning of the selected thresholds. According to data presented, the set of parameters chosen as default come up from the experiment 'Test2', which provides the best accuracy (89%), considering its values of sensitivity (84%) and specificity (94%). By analyzing in details the error accomplished in experiment 'Test2', the origin of false positive and false negative results must be attributed to the misleading view over the tissue caused by the cutting angle on the sample. In fact, a limiting factor in distinguishing the disease-affected areas from the not-affected ones concerns the tissue-cutting plane. Since it may intersect the tubule at variable angles, the resulting image does not in all cases clearly reveal the typical tubular morphology. For instance, if the plane runs perpendicularly to the tubule, all the information related to the presence of two layers of cells surrounding the lumen is preserved. On the other hand, if the tissue has been cut along the axis of the tubule it will result in a loss of morphology data (Figure 7). Unfortunately this cannot be avoided and an automatic approach can present difficulties in coping with it.

**Table 1:** Values of True Positive(TP), False Positive(FP), False Negative(FN), True Negative(TN) results and related accuracy have been reported for the four cross-validation tests.

Test	TP	FP	FN	TN	Accuracy
Test1	503	43	125	625	87
Test2	541	37	101	617	89
Test3	497	39	137	623	86
Test4	516	33	143	604	86

Beside the main dataset the algorithm has been tested with two other datasets, in order to test its flexibility while providing as input images which present varied resolution and different pixel intensity. Data about these experiments are shown in Table 2.

**Table 2:** Values of True Positive(TP), False Positive(FP), False Negative(FN), True Negative(TN) results and related accuracy calculated for the datasets that present varied resolution (R) and different pixel intensity (PI), both when maintaining the algorithm default values and when setting finely tuned parameters values.

Test	TP	FP	FN	TN	Accuracy
R default	194	21	37	108	83
R fine tuning	169	13	26	152	89
PI default	176	20	69	95	75
PI fine tuning	127	6	27	200	90

For what concerns the resolution test, five images have been used. The test performed using default parameters resulted in 83% of accuracy, 83% of sensitivity and 83% of specificity. The output of the test involving the fine tuning (obtained by modifying manually the default resolution value with the real one, thus avoiding the algorithm image resizing step) retrieved an accuracy value of around 90%, a sensitivity of 85% and a specificity of 94%. Algorithm performance is better when enabling fine tuning than when using default parameters. Even regarding the pixel intensity test five images have been used. The test performed with default parameters provided an accuracy value of 75%, a sensitivity value of 71% and a specificity value of 82%. The test which foresees the parameters fine tuning (obtained by modifying manually the default pixel intensity value with the real one, thus avoiding the algorithm color normalization step) provided an accuracy of 90%, a sensitivity of 82% and a specificity of 97%. Even for what concerns software flexibility about pixel intensity, this second test showed better performances than the previous one which implies the use of the default parameters values.

In conclusion, the algorithm shows to be robust since parameters vary slightly in the cross-validation. Moreover, the software presents good flexibility whereas it works receiving images of different resolution and pixel intensity as input. Finally, the algorithm shows a high level of adaptability, due to the possibility given to users to exploit information about the dataset images to set the correct parameters and to optimize the performance.

The time performance primarily depends on the image size: typical size range goes from 3MB to 8MB. It must be noticed that the time spent to cut the global image in a set of sub-images, and the mirror phase of resembling, have to be considered as an intrinsic sequential part of the code, while the rest of the code (consisting in independent sub-image analysis) is parallelizable. Cutting times go from 45 s to 57 s, while reassembling times go from 63 s to 125 s, according to image size. Average sub-image processing time is 49 s, with a σ = 15 s, which takes into account not only the image size but even the variability of the last algorithm step.

### A real example

In the following part an example is presented. The whole image was acquired with the previously defined procedure. The result is an image as wide as one third of the whole slide, with pixel dimensions of 6720 × 4200. Using a suitable grid dimension the image was divided into 72 sub-images.

**Figure 8:**
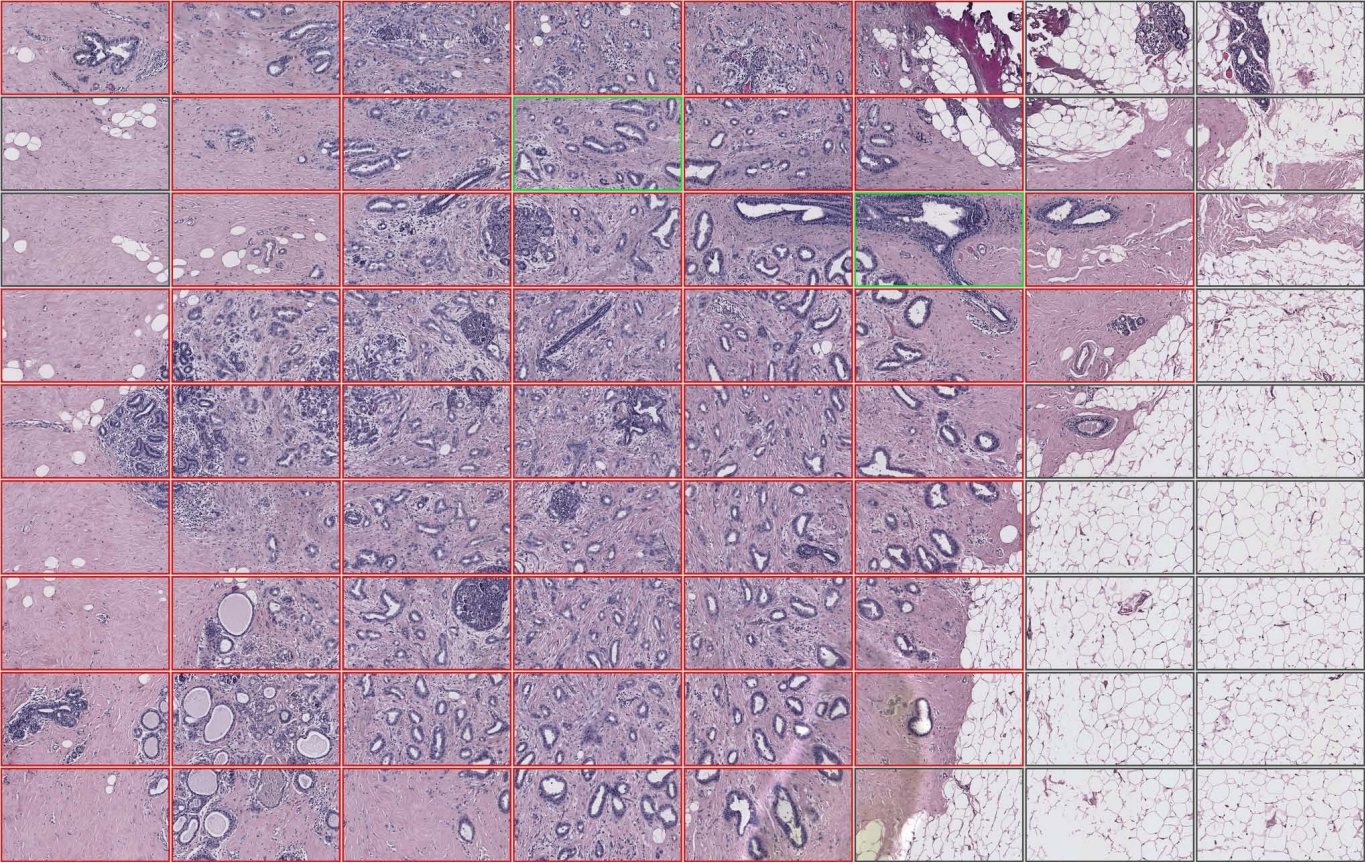
**Work flow output**. The figure shows the composite example image with the final result. As an easy reference, each sub-image is identified through its 'coordinates', given by the number of the row and the number of the column, to facilitate the output discussion. The areas shown in red are the affected ones; in case of unaffected areas they have been bordered in green. The gray areas represent non interesting zones, most of the time containing only fat cells, not useful in TMA cancer-oriented experiments.

**Figure 9:**
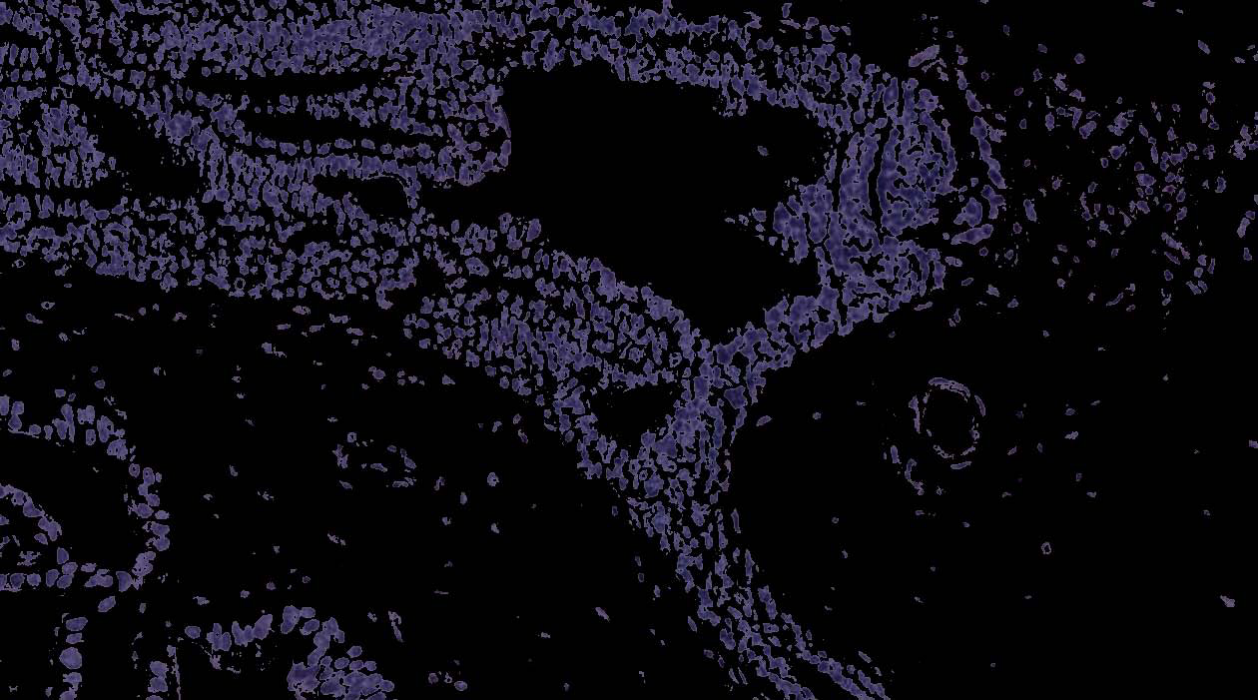
**Segmentation perspective of a tissue detail**. Images 36 as it appears after the segmentation step. It is interesting to notice the multiple layers of cells that appear in this area and that result in the unaffected evaluation.

The pipeline is applied to each sub-image using the parameters and the thresholds described above. Finally all the sub-images are collated, enriched with the computed information, as shown in Figure 8. Observing the whole output image it can be noticed that two sub-images (24: row 2, column 4; 36: row 3 column 6) are identified as normal (green line) although inside a tumour area. Concerning image 36 the software correctly identifies a region of normal tissue within the tumour area, as reported in Figure 9. This is crucial in the TMA experiments punching phase since the random choice of this area of the tissue would lead to a mistake in the data analysis.

On the other hand, the classification of image 24 among the normal ones has to be considered a false positive, enforcing the idea that the implemented software represents a first screening and support for the pathologist and therefore should be followed by human evaluation.

## Conclusions

**Figure 10:**
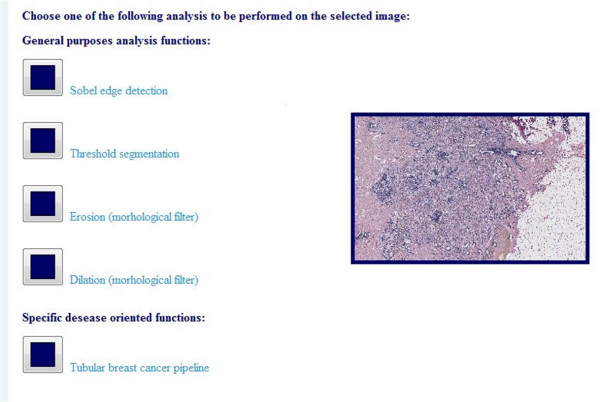
**Web page**. Web page of the TMARepDB site for accessing the tubular breast cancer pipeline of analysis.

The pilot experiment described here, involving TMA support tools, shows that it is feasible to design a program able to distinguish areas in a tissue slide as affected, unaffected and non-informative and localized them. It is currently integrated into the web based TMARepDB database, together with some other basic image processing methods, like Sobel edge detection filter and dilation morphological filter (Figure 10). This software has been developed to be used in the pre-array experiment step where, after the collection of donor blocks, affected and unaffected areas need to be identified to create the recipient block with the matrix of tissue cores. This normally needs the support of a pathologist, who marks the glass slide with a coloured waterproof pen where the areas of interest are to be found. As a first step toward process automation slides must be digitalised, in order to be suitable for storing and for using as an overlay on the donor block image, in the semi or automated TMA software. The process is further automated with software capable of distinguishing areas of interest within tissues. The generated images may be used to extract the tissue cores by overlapping the image with annotations, through computer software over the image of the tissue in the paraffin block.

The aim of this work is to show that, despite the fact that automation in this context is a very challenging task due to morphological complexity of human tissues, the implementation of a pathologist's support tool, in the TMA context, is feasible and the product is reliable. The strength of the computerized method presented here is that once it has been developed and fine-tuned it may be used to analyze numerous images in parallel in a short time.

Future developments mainly concerns the possibility of switching the final evaluation of a sub-picture, that should be given to the user to enable modifying the process output. This would result in a completely reliable evaluation of the image, which could be further overlapped to the donor block to map the punching areas on it, with the possibility of performing automatic cores picking. In the authors' plan the described application could be part of a fully TMA analysis pipeline: after the tissues collection and the staining process, the procedure would be carried out by automatic or semiautomatic arrayers, guided by software able to detect suitable punching areas, thus allowing a fast tissue microarray building.

In conclusion, although the algorithms can only approximate human competencies and experience, the developed tool represents an important aid for scientists through automation of a laborious and time-consuming job.

## Availability and requirements

Material is available at URL: ftp://fileserver.itb.cnr.it/federica/BMCTub

Software is archived in BMCTub.tar.gz; requirements and documentation are provided in the README file; the exploited datasets are contained in folder Datasets.

## List of abbreviations

TMA: Tissue MicroArray; H&E: Hematoxylin-Eosin; SVM: Support Vector Machine; VM: Virtual Microscope.

## Competing interests

The authors declare that they have no competing interests.

## Authors' contributions

FV and IM designed and implemented the algorithm, MT and MdB gave the biological perspective, FB, PR and LM supervised the work from biological and technical points of view. All authors read and approved the final manuscript.
